# Co-Creating Strategies and Recommendations to Enhance the Physical Activities of Undergraduate Students at a South African University: A Social Ecological Approach

**DOI:** 10.3390/ijerph22121803

**Published:** 2025-11-28

**Authors:** Chanté Johannes, Nicolette V. Roman, Sunday O. Onagbiye, Simone Titus, Lloyd Leach

**Affiliations:** 1 Sports, Recreation, and Exercise Science Department, University of the Western Cape, Cape Town 7535, South Africa; sonagbiye@frederick.edu (S.O.O.); titusdawsons@sun.ac.za (S.T.); lleach@uwc.ac.za (L.L.); 2 Centre for Interdisciplinary Studies of Children, Families and Society, University of the Western Cape, Cape Town 7535, South Africa; nroman@uwc.ac.za; 3 School of Biological, Physical, and Health Sciences, Health Profession Career Community, Department of Health Science, Frederick Community College, Frederick, MD 21701, USA; 4 Department of Health Professions Education, Faculty of Medicine and Health Sciences, Stellenbosch University, Cape Town 7505, South Africa

**Keywords:** co-create, physical activity, strategies, recommendations, undergraduate university students

## Abstract

Engagement in physical activity (PA) has been considered to be influenced by multiple psychosocial factors (such as mental health, motivation and social support). However, current interventions often neglect these complex interactions. The Social Ecological Model (SEM) highlights the need for context-specific strategies addressing individual, social, environmental, and public engagement domains to enhance student PA. Therefore, this study aimed to develop strategies and recommendations to enhance the PA levels of undergraduate university students by using a SEM Approach. A co-creative consensus workshop, held between July and August 2024, was employed. The stakeholder group (*n* = 25) comprised undergraduate university students, professors and academic doctors specialising in PA and health-related disciplines. Data generated from the workshop were uploaded into Atlas Ti. V8 and thematically analysed. The co-created strategies underscore the importance of a multi-level approach to enhancing PA participation among undergraduate students. A total of 32 strategies were developed, reflecting the interconnected influence of psychosocial factors across the levels of the SEM. These include strategies related to PA (9), mental health (7), motivation (9), and social support (7). By developing context-specific strategies and recommendations that address individual, social, physical, and public engagement levels, the research offers practical, student-centred solutions to enhance PA participation. The co-created strategies hold the potential to foster long-term behavioural change and promote active, healthier lifestyles within the South African university context.

## 1. Introduction

Physical activity (PA) is associated with a reduced risk of all-cause mortality, cardiovascular disease, type-2 diabetes, hypertension, breast cancer, colon cancer, gestational diabetes, ischemic heart disease, and ischemic stroke [1,2]. Physical activity has also been associated with a range of psychological health benefits, including reduced risk of depression, anxiety and stress, as well as improved mood, motivation and social support [3]. The World Health Organisation (WHO) suggested that young adults, such as undergraduate university students, do at least 150 min per week of moderate intensity aerobic PA, or at least 75 min of vigorous intensity aerobic PA throughout the week, or an equivalent combination of moderate and vigorous intensity activities [4]. However, inadequate levels of PA among this population have been consistently reported across global contexts [5,6]. In response to the widespread issue of sedentarism, previous studies have explored various strategies aimed at enhancing PA levels among university students.

A review by García-Álvarez and Faubel [7] identified eight key strategies and nine data collection tools to enhance PA among university students, with validated questionnaires as the most common assessment method. Educational health promotion, delivered online or in-person, was the most frequently used strategy. Despite promising outcomes, the review highlighted limitations, including a small number of studies, methodological differences across contexts, and follow-up challenges [7]. Similarly, Johannes et al. [8] found that interventions using social media, mobile apps, web platforms, and in-person sessions were effective in increasing PA engagement. The use of digital tools, especially social media, was emphasised as a best practice aligned with student trends [8]. However, while both reviews offer valuable insight into intervention strategies, it does not adequately address the psychosocial factors (mental health, motivation and social support) that shape PA behaviour among students. Additionally, they fall short in considering the broader contextual environments (such as individual, social, environmental, and public engagement dimensions) that shape PA behaviour.

To understand this PA decline, researchers have studied psychosocial determinants of health that influence PA engagement, such as mental health [9], motivation [10] and social support [6]. Previous research has indicated that the mental health of university students is considered among the most pressing public health areas in South Africa. For instance, the past 5 years have seen unprecedented incidents of suicide acts and attempts among university students across the country [11]. The increasingly tragic events on campuses, associated with mental health, have led many experts to suspect that we may be experiencing a mental illness epidemic among university students [12]. This suggests that supportive efforts focused on intervention programmes may be helpful [13]. Nevertheless, research showed that the motivation to exercise barriers and exercise preferences were significantly associated with PA levels [1,14]. Physical activity (PA) habits are a key factor in lifelong health behaviour among college students and a direct driver of their motivation. The acquisition of external social support can cause the PA habits of college students to be successfully generated [15]. Therefore, PA interventions for university students should consider the different possible sources and types of social support [16]. While it is essential to consider psychosocial factors such as mental health, motivation, and social support in shaping PA behaviours, it is equally important to account for the broader contextual environments from which these factors emerge and interact. One way of understanding these layered influences is through the Social Ecological Model (SEM).

### Social Ecological Model Approach

**Figure 1:**
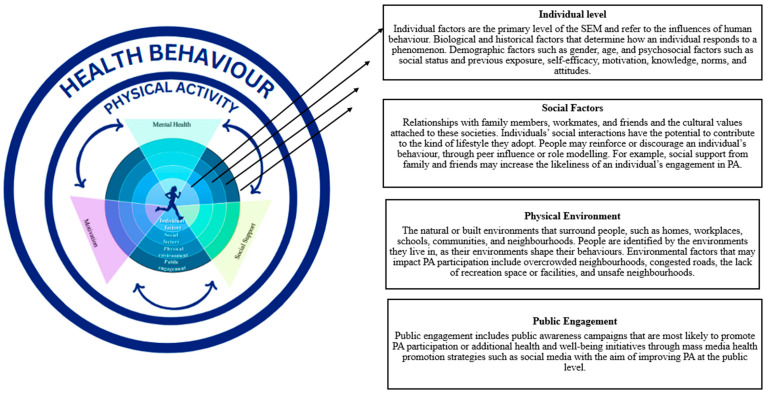
Framework of the SEM to incorporate psychosocial factors that influence PA health behaviours [19].

This study utilised the SEM as a conceptual framework as it is a comprehensive approach that considers multiple levels that influence behaviour [17,18]. This model comprises four levels, namely, individual factors, social factors, the physical environment and public policy. However, previous research has considered the term public engagement more applicable since public policy has been driven by technology [19]. Previous authors have indicated that technological sources that enable mass media communication practices, such as social networking sites, play an influential role in PA participation, as this ultimately influences what young adults view and their corresponding actions in terms of health behaviour [1,20]. Hence, for the purpose of this study, public policy was revised to public engagement on the basis that social media has been reported as a critical factor in shaping and disseminating policies, creating public health promotion and awareness campaigns as well as influencing public opinion [21]. Creating effective PA and context-specific strategies for undergraduate university students requires a holistic approach that not only considers the physical aspects of an individual but also the psychosocial factors that influence decision-making and behaviour as well [3,22]. Incorporating psychosocial factors such as mental health, motivation and social support in PA strategies according to the SEM approach is vital for placing focus on the interconnectedness of various factors that influence PA participation [23,24]. By integrating psychosocial factors into the SEM framework, the strategies become comprehensive and effective in promoting sustained PA engagement [25]. The adapted version of the SEM (Figure 1) delineates four interrelated levels of influence: individual, social, physical environment, and public engagement. Central to the model is PA, depicted as an encompassing construct that is both shaped by and reflective of broader behavioural determinants. PA engagement is not solely a function of personal choice but rather the outcome of complex, dynamic interactions among key psychosocial factors, namely, mental health, motivation, and social support. These factors operate synergistically across SEM levels to influence students’ behavioural patterns, underscoring the multifactorial and context-dependent nature of PA participation within the undergraduate university population [19].

Despite the growing body of literature on PA interventions among university students, many existing studies have predominantly focused on isolated strategies or single-level determinants, often neglecting the complex, interrelated factors that influence PA behaviour [17]. Previous research has rarely explored how psychosocial factors such as mental health, motivation, and social support operate within broader environmental and structural contexts [26]. Moreover, there is limited evidence of studies adopting a holistic framework that integrates multiple levels of influence to guide the development of PA strategies. This study addresses these critical gaps by using the SEM as a guiding framework to develop context-specific strategies and recommendations aimed at enhancing PA levels among undergraduate university students. This paper builds on a previous systematic review of global PA best practices [8] by contributing new empirical data co-created with stakeholders using the SEM framework. Its novelty lies in the incorporation of diverse perspectives from stakeholders, while directly addressing the psychosocial realities that shape student behaviour. As such, this research offers a more comprehensive and contextually grounded approach, making it a relevant and timely contribution to the field of health promotion and student well-being in higher education. Thus, there is a critical need for PA interventions in the university context that are not only multi-level but also explicitly designed to address these acute psychosocial challenges, for which a co-creative, SEM-based approach is ideally suited. Therefore, taking into consideration these factors and gaps in the literature, this study aimed to develop strategies and recommendations to enhance the PA levels of undergraduate university students by using a SEM Approach. This adapted Social Ecological Model provides a contemporary and comprehensive framework, aligning the study with current research while guiding the development of effective, multi-level strategies to promote physical activity among undergraduate students.

## 2. Materials and Methods

### 2.1. Development of the Strategies

Prior to the commencement of the co-creative consensus workshop, preliminary, evidence-based strategies derived from the foundational systematic review [8] and a mixed-methodological approach employing a sequential explanatory design were implemented to establish a robust foundation for conceptualising the workshop and to elucidate the PA levels, preferences, and psychosocial determinants among undergraduate university students [26]. In Stage 1, quantitative data were collected via questionnaires administered to a cohort of 534 undergraduate students [3], while Stage 2 involved in-depth qualitative interviews with a purposively selected sample of 18 participants. The empirical insights garnered from this dual-phase investigation critically informed the formulation of evidence-based PA strategies, which were subsequently refined and validated during the consensus workshop to ensure their applicability and efficacy within the target demographic. While these strategies were previously introduced in a previous study that focused primarily on the methodological approach used to co-create them (formally referred to as guidelines) [19], the current study shifts the focus to a detailed description of the strategies themselves, along with actionable recommendations.

### 2.2. Study Design

The consensus workshop constituted a co-creative, qualitative research process, recognised in the literature as an important approach for engaging stakeholders in collaborative inquiry [27]. This design supports critical interrogation of proposed ideas, encourages equitable participation, and mitigates power imbalances by ensuring that no single voice dominates the deliberations. Through structured dialogue, guided facilitation, and repeated opportunities for refinement, the workshop format is able to generate a shared understanding of priorities and reach agreement on key strategies. This innovative participatory method facilitates structured group discussions, ensuring that all participants actively contribute and collectively shape the outcomes of a unified dialogue. The consensus workshop framework used in this study follows an iterative, multi-round structure that enables participants to move from individual reflection to collective synthesis. Following the workshop, discussions were systematically analysed and synthesised [27]. This method was specifically chosen for the present study to achieve consensus among stakeholders, critically evaluate the proposed strategies, and ascertain their contextual relevance, validity, and alignment with the study’s objectives. A notable strength of this approach is its capacity to preserve anonymity, enabling stakeholders to express their perspectives openly and candidly without social pressures, thereby enhancing the integrity of the deliberative process [28]. The multi-round structure strengthened the rigour of the process by providing opportunities for revision, validation, and re-assessment of stakeholder input until consensus was achieved.

### 2.3. Study Setting and Participants

This study employed a purposive sampling strategy, engaging stakeholders from a single university in South Africa’s Western Cape Province to ensure depth and contextual specificity. Participants included undergraduate students and expert scholars spanning disciplines such as PA, public health, sports sciences, psychology, and student support services. By focusing on a singular institutional setting, the research facilitated a comprehensive exploration of university-specific dynamics, thereby amplifying the contextual relevance and strategic applicability of the findings. The current research adhered to established consensus workshop methodologies, which suggest an optimal participant range to balance inclusivity with deliberative efficacy. While research posits that such workshops may accommodate up to 40 stakeholders [27], empirical evidence [28] indicates that larger cohorts often diminish feedback quality and consensus coherence. To mitigate this, the current study limited participation to 20 stakeholders, ensuring robust engagement while preserving the co-creative integrity of the workshop process. Undergraduate university students were included based on the following criteria: of all genders who were registered for degree programmes at a university in the Western Cape Province of South Africa and aged 18 years or older. Students registered for non-degree purposes, enrolled for only one semester, under the age of 18, or did not provide consent were excluded. Additionally, academic doctors and professors based in South Africa with expertise in PA, sports science, or public health were included and provided informed consent. Experts outside South Africa, those without relevant specialisation, or those who did not consent were excluded.

The study utilised a two-round co-creative consensus workshop designed to iteratively refine strategic insights through collaborative stakeholder engagement. In round 1 (initial stakeholder engagement), the primary research facilitator invited twelve (12) academic stakeholders from a Western Cape university via email, ensuring alignment with the study’s inclusion criteria regarding professional expertise. The sample size accounted for potential attrition while maintaining methodological rigour. Two recruitment emails were issued, yielding participation from eight (8) stakeholders, who contributed to the co-construction of preliminary strategies. In round 2 (expanded stakeholder involvement), to enhance the robustness and inclusivity of the co-creative process, an invitation was sent to a broader participant pool, involving thirty (30) academic physicians and professors, alongside twenty-two (22) undergraduate students. Five (5) email invitations and reminders were distributed to optimise engagement. Seventeen (17) stakeholders voluntarily participated, further refining the emergent strategies through structured deliberation. Across both rounds, the co-creative workshop engaged a total of twenty-five (25) stakeholders, ensuring diverse perspectives in the development of contextually grounded strategies.

### 2.4. Data Collection Process

The consensus workshop was conducted online in English, the institution’s primary language of instruction, and comprised two rounds: Round 1 in July 2024 and Round 2 in August 2024, each lasting approximately 3.5 h. Hosted via Google Meet to engage with stakeholders irrespective of where they were located, the sessions were audio-recorded with participants’ consent. Following Charlton’s [27] structured consensus workshop framework, the primary researcher facilitated the proceedings, beginning with an introductory overview of the workshop’s aims and procedures. An icebreaker activity, in which participants defined PA in one word, was employed to stimulate engagement, establish a collaborative atmosphere, and allow the facilitator to gauge collective perceptions of PA [29]. Stakeholders were then directed to a shared Google Excel spreadsheet containing draft strategies, which they reviewed and debated within themed breakout rooms (4–5 participants per room): Room 1 addressed PA, Room 2 mental health, Room 3 motivation, and Room 4 social support. A technical assistant ensured seamless breakout room operations. The facilitator synthesised stakeholder suggestions, merging overlapping themes and mediating discrepancies to foster consensus. Voting was conducted via the spreadsheet, with participants selecting ‘Agree with the strategy’ or ‘Disagree with the strategy’; in Round 2, consensus was achieved when ≥75% of responses endorsed a strategy [30,31]. Post-workshop, transcripts were emailed to participants for verification, with three reminders issued over 15 days to confirm accuracy or solicit additions. The finalised strategies, ratified through this process, informed the subsequent data analysis phase.

This study adopted an inductive thematic analysis approach to explore qualitative data, enabling the identification, analysis, and organisation of patterns and relationships within the dataset [32]. Thematic analysis was deemed suitable as it facilitated the extraction of meaningful insights aligned with the study’s objectives. Interview recordings were transcribed verbatim by the lead researcher and imported into ATLAS. Ti (Version 8) for qualitative data management. Thematic development followed Constas’ [33] and Vaismoradi et al.’s [34] four-phase framework: initialisation, construction, rectification, and finalisation. During the initialisation phase, transcripts were thoroughly reviewed, with significant meaning units highlighted, coded, and abstracted, accompanied by reflective memo-writing to capture analytical insights. The construction phase involved data classification and comparison, where emergent codes and themes were labelled, defined, and contextualised. In the rectification phase, iterative cycles of immersion and distancing were employed to refine interpretations, ensuring thematic coherence and alignment with established scholarship. Finally, the finalisation phase synthesised the thematic findings into a cohesive narrative, integrating them with existing literature to advance understanding of the phenomenon under investigation. Themes and subthemes were continuously read, updated and merged in terms of similarity. Thereafter, the final themes were agreed upon by the primary and co- researchers [33,34].

### 2.5. Trustworthiness

Qualitative research must uphold rigorous standards of validity and trustworthiness to maintain ethical integrity and methodological reliability [35,36]. Grounded in the framework of Lincoln and Guba [37], trustworthiness is operationalised through four key criteria: credibility, transferability, dependability, and confirmability. Each criterion was systematically addressed in this study through deliberate methodological strategies [37]. Credibility was established through methodological triangulation, incorporating multiple data sources and analytical approaches to corroborate findings [38]. Additionally, member checking was employed, wherein participants reviewed transcriptions, descriptions, and preliminary interpretations to verify accuracy. Participants were provided a two-week period to provide corrections or feedback, ensuring the fidelity of the data representation [39]. To enhance transferability, the study employed thick description [40], providing exhaustive contextual details regarding the research setting, participant demographics, and methodological procedures. This approach enables readers to evaluate the applicability of findings to analogous contexts or populations [41]. Dependability (or consistency) was ensured by anchoring interpretations in the data itself, rather than relying on researcher predispositions [36]. The analytical process was rigorously documented, allowing for reproducibility. Confirmability was achieved through reflexivity and transparency [40]. Researchers explicitly acknowledged their epistemological positions and potential biases while maintaining a comprehensive audit trail. This trail documented all analytical decisions, procedural iterations, and data management protocols, permitting external scrutiny and verification [37].

### 2.6. Ethics Considerations

Ethical approval and permission from the university were obtained prior to the commencement of the study (Reference No: HS21/10/24). All stakeholders who voluntarily participated in the consensus workshop provided informed consent and signed a confidentiality agreement. At the start of the workshop, stakeholders were reminded that participation was voluntary and that they could withdraw at any time without penalty. To ensure confidentiality and anonymity, participants’ names were replaced with pseudonyms.

## 3. Results

### 3.1. Demographic Information of Stakeholders

Round 1 engaged eight institutional staff members, predominantly female (*n* = 5). Diverse professional roles spanned multiple disciplines: social work, health informatics education, student-athlete support, sports psychology, child and family studies, health professions education, medical biosciences, and sport management. Round 2 involved eight undergraduate students, again with a female majority (*n* = 5), primarily from the Community and Health Sciences faculty (*n* = 6) and in their third academic year (*n* = 5). This student cohort represented diverse academic specialisations including commerce, law, sports, recreation and exercise sciences, social work, and physiotherapy. Additionally, the second round included nine staff participants (female, *n* = 6). Their interdisciplinary expertise encompassed physiotherapy, sports sciences, sports management, biokinetics, recreation and leisure studies, disability studies, high-performance sport, exercise physiology, sports sociology, and mental/public health.

### 3.2. Overview of Strategies

**Figure 2:**
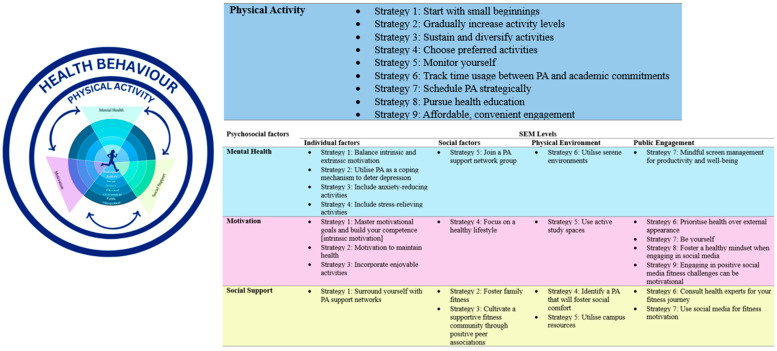
Strategies to enhance the physical activities of undergraduate university students according to the SEM.

Utilising the SEM, the current study developed a comprehensive set of strategies to enhance PA among undergraduate students, spanning four domains (Figure 2): PA, mental health, motivation, and social support. Key strategies encourage students to “start small,” gradually increase engagement, diversify activities, choose preferred exercises, and strategically schedule and track PA, while utilising accessible campus resources. Mental health strategies emphasise supportive environments, using PA as a coping mechanism, fostering intrinsic and extrinsic motivation, and mindful screen use. Motivational strategies focus on goal-setting, health-oriented mindsets, enjoyable activities, and positive social media influences. Social support strategies highlight the role of peers, family, faculty, and institutional networks in sustaining activity. Across domains, cross-cutting themes underscore the importance of practical guidance, psychosocial support, and context-sensitive approaches to promote sustainable, student-centred active lifestyles.

### 3.3. Physical Activity Strategies

Table 1 provides a comprehensive overview of strategies designed to enhance PA participation among undergraduate university students, organised according to the levels of the SEM. The table categorises strategies into four domains: PA, mental health, motivation, and social support. Each domain includes targeted interventions at the individual, social, physical environment, and public engagement levels. A total of 32 strategies are presented, offering practical, student-centred approaches that address both personal and environmental factors influencing PA.

Table 1 (created by the primary researcher) presents nine PA strategies developed through a consensus-driven, stakeholder-informed process. The table positions PA strategies as the overarching approach for promoting active lifestyles among university students. These strategies focus on starting small, gradually increasing PA engagement, diversifying activities, choosing preferred activities, monitoring, tracking time utilization, scheduling PA strategically, pursuing health education and accessing affordable and convenient engagement locations.

Table 2 (constructed by the primary researcher) outlines seven mental health strategies designed to support PA engagement among undergraduate students. Each strategy is categorised according to the SEM, demonstrating how mental health can be addressed across multiple levels of influence. The strategies emphasise the importance of creating psychologically supportive environments to facilitate sustained PA. These strategies focus on intrinsic and extrinsic motivation to change students’ mindsets, PA as a coping mechanism, support networks, utilising serene environments and being mindful of screentime.

Table 3 (created by the primary researcher) features nine motivational strategies aimed at enhancing students’ drive to participate in PA. These are structured within the SEM to reflect various sources of influence, from personal beliefs to broader community messaging. The strategies are designed to activate both intrinsic and extrinsic motivation in a context-sensitive manner. These strategies include motivational goals, maintaining health, incorporating enjoyable activities, focusing on a healthy lifestyle, utilising active study spaces, focusing on health over appearance, health mindsets, and identifying positive social media influences.

Table 4 (constructed by the primary researcher) highlights seven social support strategies that facilitate PA engagement. The strategies are mapped across the SEM, capturing key support systems such as peers, family, faculty, and institutional structures. These strategies show the value of connectedness and interpersonal encouragement in sustaining active behaviours. These strategies focus on PA support networks, incorporating family fitness, identifying positive peer associations, social comfort, utilising campus resources, consulting health experts and focusing on positive fitness motivation on social media.

## 4. Discussion

This study aimed to develop strategies and recommendations to enhance the PA levels of undergraduate university students by using a SEM Approach. The co-created strategies underscore the importance of a multi-level approach to enhancing PA participation among undergraduate students. A total of 32 strategies were developed, reflecting the interconnected influence of psychosocial factors across the levels of the SEM. These include strategies related to PA (9), mental health (7), motivation (9), and social support (7). Importantly, the strategies illustrate the value of integrating psychosocial considerations into PA promotion, emphasising that enhancing motivation, fostering supportive social networks and addressing mental health are critical components for sustaining active behaviours among undergraduate students [42,43,44,45]. In alignment with previous research, these findings provide a comprehensive, context-specific framework for universities seeking to implement effective interventions that promote holistic well-being through increased PA participation [19,46,47].

Globally, physical inactivity has been considered a growing public health concern and the fourth leading cause of mortality [48,49]. Regarding international practices, the strategies from this current study are similar to those presented in the PA Guidelines for Americans, 2nd Edition, released in 2018 [50]. This framework is the authoritative federal guidance on PA for individuals aged 3 years and older [50]. The focus of the American version of the PA guidelines was research-based, centering on health benefits related to brain well-being, active adults, risks of sedentary behaviour and long-term benefits of the emotional state of individuals. The current strategies content from this study aligns with the American PA framework. These strategies attempt to provide a solution based on co-creative collaborations among stakeholders. Therefore, the strategies proposed in this study are specifically tailored for undergraduate students in the South African context, addressing the critical need for improving health and well-being within the university environment.

Similarly to previous studies, using the SEM as a conceptual framework to enhance PA among students has been proven to be a comprehensive approach for understanding holistic well-being [17,19]. Therefore, the development of the context-specific PA strategies from our study is aligned and theoretically grounded to support undergraduate students by addressing the multiple layers of influence on their PA behaviour, including individual factors, social factors, the physical environment and public engagement [51]. Furthermore, this theory posits that a student’s behaviour is shaped by a dynamic interplay of psychosocial factors that operate at various levels within the SEM [52,53,54]. Thus, each psychosocial factor at every level of the SEM must be considered in order to effectively promote and sustain behavioural change such as enhancing PA levels [17]. As such, the development of the strategies from the current study takes into account the various psychosocial factors at each level of the SEM, ensuring a comprehensive approach that addresses individual needs, fosters social support and creates an environment conducive to enhancing PA among students [3].

Furthermore, the findings derived from this study emphasise the alignment of the context-specific PA guidelines with several Sustainable Development Goals (SDGs) [55]. Results from this study are aligned with previous authors focusing on the psychological aspects of university students’ health [55,56]. By incorporating psychosocial factors such as mental health, motivation and social support, the guidelines directly contribute to achieving SDG3: Good Health and Well-Being, by promoting both the physical and mental health of undergraduate students. These strategies recognise that well-being is multidimensional, acknowledging that PA alone is insufficient without addressing the psychological and social contexts that influence students’ health behaviours. Secondly, the research-based guidelines support SDG4: Quality Education, by fostering a campus environment that encourages not only academic success but also the holistic development of students. By integrating structured PA with strategies that enhance motivation and social connectedness, the guidelines create conditions conducive to sustained learning, engagement and academic achievement.

Thirdly, by promoting inclusivity, equitable access to PA opportunities and reducing health disparities, these context-specific guidelines contribute to SDG10: Reduced Inequalities. In conjunction to previous research [55,57], these strategies ensure that students from diverse backgrounds, including those historically marginalised or facing socio-economic barriers, can participate in activities that enhance their well-being. Fourthly, at the broader community level, the guidelines support SDG11: Sustainable Cities and Communities, by advocating for healthier and more accessible campus environments. By creating spaces that encourage active lifestyles and social interaction, the guidelines contribute to sustainable, health-promoting infrastructures within the university setting.

Lastly, by actively involving key stakeholders, including students, faculty and community partners, in co-creating and implementing the guidelines, the initiative supports SDG17: Partnerships for the Goals [58]. Previous research suggested that cross-sector and multidisciplinary partners are essential for promoting PA [58]. Thus, this collaborative approach in the current study ensured that the strategies were contextually relevant, feasible and reflective of diverse perspectives. Moreover, the co-creative method employed in this study aligns closely with principles of participatory action research [59] and empowerment theory [60]. By actively involving students, faculty, and other stakeholders in the development of PA strategies, the process fostered a sense of ownership and agency among participants. This participatory engagement not only facilitated the identification of contextually relevant strategies but also functioned as an intervention in itself, enhancing motivation and commitment to implementation. The collaborative approach thus strengthens the likelihood that the strategies will be adopted and sustained, demonstrating that co-creation can be both a method and a mechanism for behavioural and environmental change.

In terms of utilising the SEM for this current study, the adapted ‘Public Engagement’ level highlights the value of actively involving the broader community in promoting PA [17]. This innovation reflects the modern reality of health promotion, where engagement extends beyond policy and institutional frameworks to include digital platforms, social media, and community-driven initiatives [17,19]. By incorporating public engagement, the model captures real-world influences on behaviour and differentiates itself from traditional, policy-centric SEM approaches, emphasising the importance of collective participation in sustaining health-promoting practices [19].

Collectively, these guidelines exemplify how integrating psychosocial factors in PA strategies not only advances the holistic health and well-being of university students but also contributes meaningfully to global sustainable development, aligning with previous studies that underscore the intersection between health promotion and sustainable development outcomes [61,62]. The findings of this study address existing research gaps by providing context-specific insights into how psychosocial factors influence undergraduate students’ engagement in PA, offering empirical evidence that complements and extends previous studies which predominantly focused on generalised or non-university populations [6,20,52], while critically highlighting both consistencies and divergences with earlier research regarding the interplay between PA and holistic well-being [9,14].

### Strengths and Limitations of the Study

This study exhibits several notable strengths in its approach to developing PA strategies for undergraduate students. The research employed the SEM as a theoretical framework, facilitating a comprehensive, multi-level exploration of factors influencing PA behaviours. The innovative co-creative consensus workshop methodology successfully engaged a diverse range of stakeholders, including both students and academic experts across relevant disciplines, ensuring the developed strategies benefited from both professional expertise and student perspectives. This enhanced the practical applicability of the resulting 32 strategies, which were systematically organised across four key domains: PA, mental health, motivation, and social support. Importantly, the study’s alignment with both South Africa’s NDP for 2030 and the United Nations SDGs demonstrates its policy relevance and potential for wider implementation. Nevertheless, some limitations should be acknowledged when considering this study’s findings. The research sample was drawn exclusively from a single higher education institution in the Western Cape province, potentially limiting the transferability of findings to other university contexts within South Africa or internationally. Future research should consider broadening similar research to additional universities, locally and internationally. Methodologically, the time-constrained workshop format, while effective for consensus-building, may not have allowed for in-depth exploration of more complex issues. Thus, researchers should explore a face-to-face workshop approach to enhance collaborative dialogue and stakeholder engagement.

## 5. Conclusions

Results from this study contribute to a deeper understanding of the multilayered factors, such as mental health, motivation, and social support, that influence PA among undergraduate students, using a SEM approach. A total of 32 strategies were co-created, reflecting the interconnected influence of psychosocial factors across the levels of the SEM. These include strategies related to PA (9), mental health (7), motivation (9), and social support (7). By developing context-specific strategies and recommendations that address individual, social, physical, and public engagement levels, the research offers practical, student-centred solutions to enhance physical activity participation. These strategies are aligned with South Africa’s NDP 2023 and the United Nations Sustainable Development Goals, supporting broader national and global efforts to improve health and well-being. The strategies have the potential to foster long-term behavioural change and promote active, healthier lifestyles within the South African university context.

## 6. Recommendations

The following set of recommendations is intended to guide a diverse group of stakeholders, undergraduate university students, health professionals, researchers, and policymakers, by providing evidence-informed, context-specific strategies to support, implement, and promote PA at multiple levels (i.e., individual, social, physical and public engagement layers). These recommendations aim to foster a more active student population, inform institutional health initiatives, and contribute to the development of sustainable, inclusive policies that align with national and global health priorities.

### 6.1. Undergraduate University Students

For undergraduate university students, the following recommendations are provided. Firstly, become vocal by actively participating in university discussions and forums related to health and wellness: use your voice to advocate for the integration of PA and wellness programmes into campus life. Provide feedback on existing health initiatives and suggest areas for improvement based on a student’s perspective. Simultaneously, participate in campus health and wellness interventions that focus on PA awareness. Secondly, lead by example by becoming health peer leaders: demonstrate the benefits of PA by incorporating health and wellness into your lifestyle. Your commitment to PA may inspire others to follow the same pursuit. Share your experiences by becoming a health peer leader. Student health peer leaders should be actively engaged and collaborate in opportunities where their voices and opinions are valued. Be a student representative and advocate for health initiatives that are suitable, appropriate and relevant to the student body. Thirdly, use technology to your advantage: university students are considered to be digitally inclined to social networking sites and additional digital platforms. Take advantage of this familiarity to enhance your PA levels and inspire your peers through similar digital channels. Communicate, seek assistance and share resources related to PA among peers. This would enhance your understanding and encourage healthy and supportive networks.

### 6.2. Health Professionals and Experts

For health professionals and experts, the following recommendations are suggested. Firstly, health professionals should strive to enhance student wellness through an interdisciplinary approach. Sports scientists, psychologists, student societies, campus clinics, and recreational administrators should collaborate and co-create initiatives that not only enhance PA participation but focus on holistic support for mental health, motivation, and social support as well. Secondly, sports psychologists may assist with behaviour change techniques to promote PA and reduce sedentarism among undergraduate students. It is recommended that future research examine the effectiveness of these collaborative and co-creation initiatives to deliver successful strategies that may address physical inactivity. Thirdly, health experts working in the context of health and PA should obtain a deeper understanding of the unique experiences and needs of undergraduate students’ sand thereby provide initiatives that are context-specific in terms of mental health, motivation and social support. Fourthly, to encourage PA participation among undergraduate students, a holistic approach is needed. This may involve collaborative efforts with support services and a focus on diversifying PA initiatives, introducing peer-led programmes to track PA levels. Next, mental health professionals could reinforce PA as a coping strategy and an outlet. Workshops could be implemented to promote the relationship between mental health and PA. Additionally, health experts should be sensitive to each student who seeks advice and would like to begin with their health journey. One size does not fit all in this case. Therefore, the guidelines may be used as a foundation for assisting students who would like to commence and enhance their PA levels. Health experts are therefore recommended to tailor these guidelines according to the students’ needs, preferences and their context. Lastly, the Fourth Industrial Revolution is permeating the realm of education and teaching and learning practices. Academics should explore innovative methods of imparting and co-constructing knowledge by using various online teaching methods. Therefore, PA initiatives should incorporate digital technology such as social media practices, as this is a convenient way of delivering visually appealing information that is relevant to undergraduate students. Furthermore, this would provide a safe space that is affordable and convenient for regular PA participation.

### 6.3. Future Research

Regarding future research, the following recommendations are provided. Further research with a larger cohort including postgraduate students is recommended. Additional universities and educational contexts within South Africa should be investigated to enhance the generalizability. Additionally, longitudinal studies may be warranted to track and monitor PA behaviours and levels of students. Researchers may consider assessing the effectiveness of innovative PA education and health promotional strategies, utilising social network sites to motivate and enhance PA levels. This could involve utilising sites that are frequently explored such as social networking sites, websites and mobile phone applications. By investigating the effectiveness of these interventions, researchers could design and assess context-specific PA campaigns tailored to contemporary students. Regarding monitoring and evaluation, research initiatives may focus on effective monitoring and evaluation procedures to measure the effectiveness of the PA intervention. Physical activity (PA) levels are known to fluctuate or decrease at various points in a university student’s journey. It is therefore crucial to monitor when PA levels fluctuate and when students are at risk of leading sedentary lifestyles. Lastly, future research should now focus on implementing and evaluating the real-world effectiveness of these co-created strategies. This includes studying the barriers and facilitators to their adoption, their impact on actual PA levels and mental health metrics over time, and their cost-effectiveness.

### 6.4. Policymakers

In terms of policymakers, the following recommendations are suggested. It is recommended that PA be prioritised at the university level by implementing PA interventions and programmes that are suitable and relevant to the contemporary student. Additionally, universities should integrate the developed context-specific PA guidelines into their existing health and wellness policies. This integration could incorporate PA into the fabric of campus life, creating incentives for participation and promoting the benefits of PA through campus-wide campaigns. Universities could incentivise participation through credit systems, integrate ‘movement breaks’ into long lecture schedules, and ensure PA is a core component of first-year orientation programmes. The Department of Health and Higher Health should utilise these guidelines as a foundational framework to develop targeted campus-based initiatives that foster collaboration between health professionals and tertiary institutions to ensure effective implementation. The Department of Higher Education and Training (DHET) should reinforce that universities should incorporate PA into the academic curriculum that not only focuses on theoretical aspects but the physical components of PA as well. This would serve as a proactive approach to enhance PA, which ultimately contributes to achieving Sustainable Development Goal 3, which focuses on ensuring healthy lives and promoting well-being. It is important to necessitate co-creation and collaborative efforts between universities and students to develop student-tailored PA initiatives that would be suitable and relevant. Thus, students should be involved in policy and mandate creation. This would provide a student perspective that reflects their everyday experiences. Having student representatives in a steering committee for advancing health within the university setting is crucial to ensuring that health interventions are student-tailored. These representatives could provide valuable insights, advocate for student interests and preferences and ultimately facilitate health programmes. This would result in a more health-conscious campus community.

## Figures and Tables

**Table 1:** Description of PA strategies according to the SEM.

SEM Level	Strategy	Description
Across all levels.	**Strategy 1: Pursue health education**	Seek opportunities to learn about your health. The campus environment hosts a number of health experts such as professors, doctors, and sports administrators. These experts would be able to provide you with guidance tailored to your personal fitness needs and goals. By increasing your understanding of PA and its importance, you can overcome barriers and make informed choices to enhance your PA. Utilise campus resources, attend seminars, and participate in workshops to continually enhance your understanding of health-related topics. By fostering a proactive approach to health education, you can cultivate lifelong PA habits.
	**Strategy 2: Start with small beginnings**	Embarking on a physical fitness journey starts with small, simple steps. Begin with low-intensity activities like walking or stretching, dedicating 10–15 min to them, 3 days a week (this takes approximately 6–8 weeks). This gradual approach allows for a manageable entry into regular PA with a low chance of injuries occurring. By starting small, you build a foundation of consistency and confidence in your ability to engage in PA regularly. These initial steps not only help condition your body but also establish a routine that can be expanded upon as your fitness level improves. Embracing this approach sets a positive tone for your fitness journey, ensuring sustainable progress towards your health and wellness goals.
	**Strategy 3: Gradually increase activity levels**	Elevate your PA regimen by boosting both frequency and duration. Aim for 2–3 days per week, such as jogging, for 30–45 min each session. Remember to start with low-intensity activities and build towards moderate-intensity cardiovascular activities. Thereafter, high-intensity activities. Incorporate progressive programs that gradually intensify over time, ensuring enjoyment to sustain motivation, perhaps transitioning from beginner to intermediate classes like air boxing. Utilise free fitness apps to track progress and stay motivated along your fitness journey. Tracking your achievements and setting new goals can help you maintain momentum and track your improvement over time. By gradually increasing your activity levels in a structured and enjoyable manner, you will build endurance, strength, and a sustainable commitment to your physical health.
	**Strategy 4: Sustain and diversify activities**	Sustain your current PA levels while incorporating new challenges to avoid plateauing. Once you have gradually built your PA levels, from being inactive to minimally active, continue to move toward more challenging activities. Aim for 4–5 days per week of vigorous-intensity exercise, such as high-intensity interval training (HIIT), lasting 45–60 min per session. Focus on diverse fitness aspects like strength, endurance, and flexibility to keep engagement levels high. This approach ensures continuous improvement and prevents monotony in your fitness routine. A balanced approach supports long-term adherence to your fitness goals.
	**Strategy 5: Choose preferred activities**	Choose endurance activities that you enjoy, such as walking, jogging, or even joining an endurance group fitness class like Zumba. For example, you can incorporate jogging into your routine by scheduling three 30-min sessions per week. Start with a brisk walk to warm up, then gradually increase your pace to a comfortable jogging speed. Focus on maintaining a steady rhythm and breathing pattern as you enjoy the outdoors or explore scenic jogging paths on campus. Jogging not only improves cardiovascular endurance but also offers a refreshing break from academic demands, leaving you energized to focus on your studies.
	**Strategy 6: Monitor yourself**	Monitoring your activity intensity using the talk test is a practical way to gauge your PA level. During light to moderate activities, such as brisk walking at a comfortable pace, you should be able to maintain a conversation easily with a peer without feeling breathless. This indicates that your fitness intensity is appropriate for sustaining aerobic endurance and overall fitness. Conversely, if you find it challenging to speak in full sentences during PA, such as during HIIT or sprinting, you are likely exercising vigorously. This higher intensity can be beneficial for improving cardiovascular fitness and calorie burning, but it may require shorter bursts of activity followed by rest periods to maintain intensity and avoid fatigue. By using the talk test, you could adjust your fitness intensity accordingly to ensure that your workouts are effective and safe. Additional methods to monitor your PA progress are to maintain an activity log using your smartphone, fitness watch, or journal. As a university student, use the International Physical Activity Questionnaire (IPAQ) or a fitness application every Sunday evening to assess your PA levels throughout the past week. This tool is valuable for monitoring how your academic schedule impacts your fitness habits, helping you adjust your exercise routine to ensure you meet the recommended PA guidelines. Regularly using the IPAQ not only promotes awareness of your health but also supports your academic performance by maintaining a balanced lifestyle. This allows you to track your progress over time, identify patterns, and set realistic goals, serving as a practical tool for maintaining optimal fitness intensity levels tailored to your objectives.
	**Strategy 7: Track time usage between PA and academic commitments**	Document how you spend your time for one week [include all academic commitments as well]. Identify areas where you are able to incorporate PA into your daily routine [utilise a time management schedule for assistance]. Break physical activities into small segments, i.e., if you have a full day of academic workload, do an activity such as jumping jacks for 5–10 min bouts, 3–4 times a day. This would be equivalent to 30 min of activity daily. Using campus stairs instead of elevators can contribute to meeting weekly activity goals without compromising academic progress.
	**Strategy 8: Schedule PA strategically**	Make PA part of your lifestyle. It is important to plan PA into your daily routines. Plan your academic schedule around evening physical activities in order to remain consistent with your fitness routine. Students can schedule their PA time by allocating specific slots in their daily or weekly calendars. For instance, designate Monday, Wednesday, and Friday evenings from 6:00 PM to 7:00 PM for PA. During these sessions, engage in endurance activities like brisk walking or running. This structured approach would ensure regular participation in PA while allowing flexibility to accommodate academic commitments.
	**Strategy 9: Affordable, convenient engagement**	Engage in cost-effective, convenient activities such as walking around campus, jogging, or aerobic activities that do not require expensive equipment. Campus gyms offer discounted prices for students. This may be a more viable option than off-campus PA resources and facilities. By choosing affordable and convenient activities available on campus, you create sustainable habits that contribute to your well-being. Downloading PA videos from social networking sites such as YouTube, Instagram, and/or Facebook in a WIFI area may be a useful strategy as it is inexpensive and convenient. This would allow you to engage in the activity anywhere and anytime that is suitable for you.

Note: PA = Physical Activity; SEM = Social Ecological Model.

**Table 2:** Description of mental health strategies across the SEM levels.

SEM Level	Strategy	Description
Individual factors	**Strategy 1: Balance intrinsic and extrinsic motivation**	Mental health begins with both intrinsic and extrinsic motivation, driving you from within and through external influences. To sustain motivation for PA, it is important to find a balance between internal satisfaction (intrinsic motivation) and external rewards (extrinsic motivation). Choose activities that you genuinely enjoy and set personal goals that resonate with you, such as improving your emotional state or gaining a sense of accomplishment. Intrinsic motivation strategies can be effectively integrated into your routine by selecting physical activities that you find fun and fulfilling, such as dancing or hiking. Engaging in these enjoyable activities regularly helps maintain interest and joy in physical fitness. Setting personal goals that are meaningful to you is beneficial for many reasons such as feeling more energetic or reducing stress and could provide a constant source of motivation. Reflecting on these goals regularly reinforces their importance and keeps your motivation high, ensuring a sustained commitment to your PA habits. Extrinsic motivation strategies could enhance your commitment to PA by incorporating elements of rewards and progress tracking. Joining a fitness class or partnering with a workout buddy is beneficial and could make physical activities more enjoyable and rewarding. Implementing a rewards system where you earn points for each workout, which can be redeemed for small cheat meals, adds an extra layer of motivation by offering tangible incentives for your efforts, thus reinforcing your dedication to maintaining an active lifestyle.
	**Strategy 2: Utilise PA as a coping mechanism for depression**	Depression is a mental health disorder characterised by persistent feelings of sadness, hopelessness and a lack of interest or pleasure in activities. It can affect a person’s thoughts, behavior and overall functioning, leading to emotional and physical concerns. Use PA as an outlet before the onset of depressive symptoms. Keep a PA journal [i.e., time, date, type of PA, how you felt] to monitor your feelings and compare differences in emotional state and energy levels. Incorporate low to moderate-intensity activities using the FITT (Frequency, Intensity, Time and Type) principle. Participating in low-intensity group activities such as walking or gentle yoga, for 30–35 min, 3–5 days a week, can help reduce symptoms of depression and encourage a sense of belonging. For more intense activities, students may participate in air boxing to improve their emotional state. This provides an outlet for pent-up anger and frustration. Boxing requires intense focus and concentration, which can help take the mind off negative thoughts and provide a mental break from worries.
	**Strategy 3: Include anxiety-reducing activities**	Anxiety is a mental health condition characterised by excessive and persistent worry, fear or nervousness about everyday situations. It may lead to physical symptoms such as increased heart rate, sweating, trembling and dizziness. Incorporate activities that reduce anxiousness and promote relaxation 3-4 days per week, at a low intensity, 30 min per session (for example deep stretching focuses on mindfulness and controlled breathing, which can be particularly effective for reducing anxiety). Moderate-intensity activities may include a movement-based type stretching which is a slow and purposeful movement with deep breathing, such as tai chi. Simultaneously, listen to music while being physically active. Depending on the type of music you prefer, this could either be relaxing or energising. This may be an outlet to reduce tension and anxiousness therapeutically.
	**Strategy 4: Include stress-relieving activities**	Stress is the body’s response to any demand or challenge, whether physical, mental or emotional. Symptoms of stress include irritability, fatigue, difficulty concentrating and disrupted sleep patterns. Use PA to connect your mind and body through constructive coping mechanisms. Aim for 30–45 min for more substantial and sustained stress relief. Intensity should be moderate to increase the heart rate (activities may include swimming and aerobic fitness classes). Brisk walking can reduce stress by increasing heart rate. Experiment with different activities and integrate stress-reducing techniques into your routine (particularly during stressful periods such as the onset of examinations). Prioritise activities that positively influence your mental well-being. Focus on activities such as yoga that you are able to do in your bedroom, that would enhance feelings of relaxation. Regularly engaging in these activities can help clear your mind, provide a mental break from academic pressures and improve your overall emotional health, to manage and cope with stress and everyday life
Social factors	**Strategy 5: Join a PA support network group**	Building or joining a community or support network may be beneficial for a student dealing with a mental illness, by enhancing motivation and making PA more enjoyable. Any type of movement that helps an individual find their rhythm and flow at a pace that feels safe for them is important. For first and second-year students building a social capacity may be tough however speaking to students who are in their third, fourth or even postdoctoral students may be beneficial for joining an established PA community. In this community, conversations need to be held around the benefits of PA for mental health. Participating in group fitness classes, such as spinning or dance, can introduce you to like-minded individuals and provide a sense of community. The social aspect and energy of the group could enhance your emotional state and motivation. Joining a local or online fitness community allows you to share your progress, receive encouragement and celebrate achievements, even if you are starting from scratch. Having a support network can improve mental health by providing a sense of belonging, reducing feelings of isolation and offering a platform to express yourself and seek help. Surround yourself with supportive individuals who share your fitness interests; engage with them regularly to stay motivated and inspired. This approach not only helps you stay active but also provides the social support crucial for mental well-being.
Physical environment	**Strategy 6: Utilise serene environments**	To enhance mental health through PA on campus, utilise serene environments such as green spaces and nature reserves. These peaceful settings provide ideal locations for activities like walking, jogging or yoga, which promote mindfulness and relaxation. Aim to engage in these low to moderate activities 3–4 times per week for 30–45 min each session. This regular commitment not only supports physical health goals but also nurtures mental resilience by offering dedicated time to unwind and reconnect with nature. Adjust the frequency and duration based on your schedule and preferences, ensuring that your PA practice remains sustainable and enjoyable. The most important part is to remain moving on a weekly basis. All forms of PA would be beneficial for health and well-being.
Public engagement	**Strategy 7: Mindful screen management for productivity and well-being**	Be mindful of potential distractions and overreliance on social media. For example, designate specific times of the day for checking social media, such as during lecture breaks or after completing a workout. Replace excessive scrolling with active breaks, such as going for a walk outdoors or stretching (as screentime causes stiff back muscles and brain fatigue). Have an alarm as a reminder to stretch and stand regularly to prevent stiffness caused by excessive screentime. By replacing passive scrolling with purposeful breaks, you could effectively manage your screen time, stay engaged in PA and foster a healthier balance between technology use and personal wellness. Maintaining a balance between PA and mental health involves setting boundaries around social media and television use. Allocate specific times each day for checking social platforms to prevent it from overshadowing your commitment to staying physically active. Consider practices such as turning off devices at a certain time to obtain mental rest, which is key for calming the nervous system and enhancing physical and mental health. By limiting screen time, you create opportunities to engage in activities that benefit both your physical fitness and mental well-being. Consider outdoor activities like walking or running, which provide fresh air and stress relief. Use social media as a tool to inspire and connect with communities focused on both PA and mental health strategies. This approach ensures that technology complements rather than dominates your daily routine, fostering a holistic approach to wellness.

Note: PA = Physical Activity; SEM = Social Ecological Model.

**Table 3:** Description of motivational strategies according to the SEM levels.

SEM Level	Strategy	Description
Individual factors	**Strategy 1: Set motivational goals and build your competence (intrinsic motivation)**	Choose activities that you will enjoy. You will be more likely to stick with it. If it is not fun, avoid it. Remember that all PA counts. Choose activities that are appropriate for your exercise capacity-so that you will feel good. Incorporate the SMART (Specific, Measurable, Achievable, Realistic and Timely) principle when setting goals. Start with achievable goals that allow you to experience accomplishment, gradually increasing difficulty as you improve. Incorporate a variety of physical activities that cater to your strengths and interests, such as strength training, flexibility activities or aerobic workouts. Track your progress weekly (via a diary, fitness application or a desk calendar) to see improvements over time, boosting confidence and motivation. Post goals, images of ideal self and notes of encouragement in a readily seen place for self-motivation. Reward yourself for working toward your fitness goals and for attaining them. For example, after a month of continuous PA, treat yourself to a special wish [i.e., it may be new running shoes or a night out with friends]. This would help you sustain motivation. Thereafter, it would help if you continued being physically active until you no longer require external rewards but rather gain intrinsic rewards for yourself.
	**Strategy 2: Motivation to maintain health**	Use the International Physical Activity Questionnaire Short Form as quick way to figure out your current PA level. This is a seven-item questionnaire that assesses PA across seven days. Thereafter, tailor your PA routine to match your current fitness levels to maintain long-term engagement. Focus on becoming ‘in the zone’ when being physically active. This is an intrinsic mental zone where you solely focus on the activity at hand. Use this to push yourself to achieve better PA results. Focus on maintaining your health by including a variety of activities that are enjoyable and according to your preference(s). Aim to improve cardiovascular fitness by incorporating aerobic exercises like running or cycling. Start with achievable goals, gradually increasing the intensity and duration, and stay motivated by tracking your progress and celebrating milestones. Avoid performing too much PA too soon as this becomes demotivational.
	**Strategy 3: Incorporate enjoyable activities**	Find activities that are enjoyable and sustain motivation. Do not start with activities abruptly as this would lead to demotivation for not obtaining desired results. Find activities that are both challenging but enjoyable according to your preferences. For instance, forcing yourself into a workout routine that feels like a chore, rather explore dance fitness classes like Zumba, hip-hop or salsa that you find enjoyable. Dance fitness classes provide a fun and energetic atmosphere where you can move to the music and express yourself while being physically active. Starting with beginner classes tailored to your skill level allows you to enjoy the activity without feeling drained, sustaining your motivation for continued participation.
Social factors	**Strategy 4: Foster a healthy lifestyle**	Maintain motivation for PA by prioritising activities that positively influence mental health and personal expectations rather than succumbing to external pressures. Embracing variety, listening to your body and celebrating every win can contribute to a sustainable and fulfilling approach to PA, fostering a healthier and happier lifestyle throughout the university journey. Stay motivated by meeting personal expectations, not external pressures. Focus on intrinsic rewards like personal growth and progress. For example, set a goal to improve running endurance by completing a certain distance or duration, rather than aiming to run faster or farther to meet others’ expectations. This enhances your sense of accomplishment and mastery. Integrating both intrinsic and extrinsic motivation strategies creates a balanced approach to PA, fostering motivation, commitment, and supporting fitness goals. Balancing personal well-being with external incentives maintains engagement and enjoyment, ensuring sustainable progress and fulfillment and ultimately fostering a healthy lifestyle.
Physical environment	**Strategy 5: Identify and utilise active learning spaces**	Using the campus environment could be beneficial for PA engagement. Using university recreational spaces such as open soccer fields, basketball courts and swimming pools are free and convenient resources. Incorporate PA into your study routine with ‘Active Learning Spaces’ on campus, such as utilising ‘walk and talk’ paths for mobile peer discussions. Choose your preferred method to integrate movement while studying in libraries, study halls and common recreational areas. By utilising these spaces effectively, you can enhance your physical well-being and academic productivity while on campus. This approach not only enriches the study experience but also cultivates habits that integrate PA with intellectual engagement, motivating students to adopt practices that could lead to long-term benefits in both academic performance and well-being.
Public engagement	**Strategy 6: Make your health as important as your appearance**	External appearance, when referring to a person, encompasses their outward look or visual presentation. This includes aspects like clothing, grooming, hairstyle, facial expressions, body language and overall physical condition. Focus on personal health goals rather than external appearance. Engage in physical activities that make you feel strong and healthy, such as strength training or running, to build confidence and improve your body shape naturally. Avoid using PA to prioritise your external appearance, instead make your health just as important. By prioritising your well-being, you will find greater satisfaction and motivation in your fitness journey.
	**Strategy 7: Be yourself**	Avoid copying other individuals. The challenge of competition could be a motivational factor; however, it may also lead to excess stress. Focus on personal progress rather than competition or ego-driven motives compared to your peers. For example, weightlifting. Instead of comparing the number of weights lifted with others in the gym, focus on your own strength and progress. Set personal realistic goals according to your fitness level. This would allow you to build your PA competence and derive motivation from your personal achievements rather than external comparisons. Remember to be yourself as this is your PA journey.
	**Strategy 8: Foster a healthy mindset when engaging in social media**	Social media can be a motivational start to your PA journey. Social networking platforms may encourage you to live a healthier lifestyle. It is important to note that when using social media, students need to be mindful of its influence on how we perceive body image and fitness. Take proactive steps to curate a positive digital environment by following accounts that promote body positivity and diversity. These accounts celebrate different body types and encourage self-acceptance, helping to challenge societal beauty norms. Additionally, prioritise accounts that emphasise mental health awareness, offering insights into self-care practices and stress management. Limit exposure to content that may trigger negative self-perceptions or unrealistic physical appearance expectations. By carefully selecting the content you engage with, you can foster a healthier mindset and cultivate a supportive online community that aligns with your values of body positivity and mental well-being.
	**Strategy 9: Engaging in positive social media fitness challenges can be motivational**	Participate in university-specific fitness challenges on social media platforms like Instagram or TikTok. These challenges can include daily step counts, workout routines, or wellness tips, fostering a supportive online community and encouraging consistent PA participation. By participating actively, posting your progress and interacting with others using designated hashtags, you not only hold yourself accountable but also inspire and receive support from others. These challenges foster creativity in your exercise regimes, provide recognition for achievements and encourage consistent participation in PA, making your fitness journey both enjoyable and rewarding.

Note: PA = Physical Activity; SEM = Social Ecological Model.

**Table 4:** Description of social support strategies according to the SEM levels.

SEM Level	Strategy	Description
Individual factors	**Strategy 1: Surround yourself with PA support networks**	Enhance your fitness journey by identifying positive support networks that foster intrinsic motivation (such as PA support groups). Surround yourself with peers and friends who are supportive, encouraging, and uplift you, particularly on days when you do not feel motivated to be physically active. It is important to set boundaries and maintain healthy relationships. Avoid negative social behaviours and peer pressure [such as excessive drinking and partying] that could negatively impact your health journey. Engaging with others fosters a sense of relatedness and belonging, providing motivation through shared experiences and mutual encouragement. Participate in group activities or take fitness classes together, as many students find motivation in spending time with others and sharing common activities. Embrace the social aspects of PA to build a sense of community and keep each other motivated on the path to better health and fitness.
Social factors	**Strategy 2: Promote community wellness**	Foster open communication and participate in physical activities together to promote extrinsic motivation through family bonding and community support. Speak to your family or community members about your health goals. Participate in outdoor games such as ‘Campus Olympics’. Each team should aim to include participants from diverse backgrounds, such as different generations or family clans, to enhance teamwork. Teams must select a name and create a list of members. The challenge begins at a designated spot on campus, where teams receive their first clue to find the next location. Along the way, teams are provided with a balloon to blow up and decorate as a team mascot. At the second clue location, teams must complete an obstacle course, which might include a crab crawl or hula hooping to retrieve their team flag. After finishing the obstacle course, teams receive the second clue leading to a war-cry competition, judged on creativity, with a 10-min time limit. Upon completion, they receive the third clue and proceed to the final activity, which could include tug of war, water balloon toss, or similar games. Throughout the competition, teams must solve and complete a treasure hunt challenge to find hidden objects, earning 20 points for each treasure found. By participating in physical activities together, you can strengthen family and community bonds, promote open communication, and support each other’s health and well-being. Convincing family and community members who are reluctant to be physically active requires patience, understanding and a supportive approach. Lead and encourage by example and integrate manageable activities into daily routines that emphasise health benefits such as improved emotional state and fitness. Encouraging community-style fitness among university students emphasises the importance of staying active and supporting each other’s well-being, contributing to a positive campus culture that values health and teamwork. Be patient and acknowledge that change requires time; offer continuous encouragement and support throughout the journey.
	**Strategy 3: Cultivate a supportive fitness community through positive peer associations**	Surround yourself with supportive friends and peers with similar fitness goals and interests and participate in group activities to maintain motivation and mutual accountability. Create an ‘Activities’ day where each week a group of peers is gathered on the campus grounds to participate in a particular activity. Activities should vary each week to maintain motivation (activities may include fitness challenges, dance parties, and obstacle courses). Another activity may include ‘Multicultural Activities Day’. This would involve hiking at the university nature reserve with stations at different locations where students could learn about various cultures. Having regular friendly competitions would build friendships and opportunities for social interactions. Regular friendly competitions and shared experiences not only build friendships but also provide opportunities for stress relief and positive emotional connections. Promote inclusivity by welcoming friends at varying stages of their wellness journey. It is important to respect and be empathetic to individuals who are beginners in PA. Offer both group activities and opportunities for solitary activities to accommodate diverse preferences. These interactions can boost self-esteem, reduce feelings of loneliness, and improve overall emotional state, contributing to a supportive and mentally enriching campus environment.
Physical environment	**Strategy 4: Identify a PA that will foster social comfort**	Choose on-campus social fitness support activities that you prefer. For example, one activity may be to engage in campus-based flag football games. Form teams with friends or classmates and schedule regular flag football matches on a designated field or open area on campus. Flag football offers a fun and challenging way to improve cardiovascular fitness and teamwork skills. Another activity may include ‘Fitness Bingo’. This involves creating bingo cards with a grid layout, filling each square with a different PA with different levels of intensity or challenge, such as jumping jacks or jogging on a spot. Distribute the cards to various individuals along with markers. Explain that participants need to complete the physical activities listed on their cards and mark off each square as they go. The objective is to achieve bingo by completing a line (vertically, horizontally, or diagonally) or filling the entire card. Announce “bingo” when a participant achieves the goal. This activity combines fun and physical exercise, motivating participants to stay active and engage with one another in a social manner. By participating in these games, students can enhance their sense of camaraderie while reaping the physical and social benefits of regular PA. It is important to ensure safety and accessibility in campus-based activities (this may be the responsibility of the university and thus students may approach and incorporate different student support units to be involved in the PA initiatives on a monthly or termly basis).
	**Strategy 5: Utilise Campus recreational or fitness resources**	Take advantage of the fitness facilities, recreational spaces, and outdoor areas available on campus such as the nature reserve, soccer fields, athletics track and swimming pools. It is important to explore your campus environment to ascertain which resources and facilities are available. This would be an inexpensive and safer option to be physically active. For instance, devise a schedule incorporating various activities utilising different facilities and resources. For example, on Day 1, engage in treadmill running at the gym or at the athletics track; on Day 2, organise a peer yoga session in an open field or aqua aerobics at the swimming pool; on Day 3, consider attending a group fitness class at the campus recreation centre. By diversifying your activities and utilising convenient resources, you can maintain an exciting and engaging fitness routine.
Public engagement	**Strategy 6: Consult health experts for your fitness journey**	Seek personalised guidance and support from health professionals such as fitness trainers and doctors/professors in the field of health and well-being, to develop and maintain a PA plan. Seek credible and accredited health professionals by speaking to student support services and departments. These departments may assist with referrals to health experts at the university. Health experts may be seen as an important form of social support, who offer personalised guidance and encouragement to enhance your PA journey. Access to on-campus health experts is typically free or available at reduced student rates, making their support more accessible and contributing to a supportive community environment. These professionals can provide expertise and accountability, helping you overcome barriers and navigate challenges to ensure the effectiveness and sustainability of your fitness journey.
	**Strategy 7: Use social media for fitness motivation**	Use social media for information. Social media may be a useful tool for enhancing PA levels. Students should seek advice and information from accredited and reputable social media pages such as the World Health Organisation, United Nations Educational, Scientific and Cultural Organization (UNESCO) or fitness influencers such as ‘Glow with Jo’, who have a wealth of expertise in the subject matter Remember to think critically and evaluate online information. To determine the credibility of health organisations or influencers, look for credentials, certifications, or accreditations that highlight their expertise in health and wellness. Reliable sources should base their advice on scientific research and evidence rather than anecdotal claims. This would enhance a student’s PA knowledge and the benefits of regular participation. For example, engage with reputable and credible fitness influencers on social media who provide workout routines, tips, and motivation. Follow their recommended activities and incorporate them into your own fitness regimen according to your fitness level. Always remember to consider your health levels-that means starting small and gradually building your fitness level.

Note: PA = Physical Activity; SEM = Social Ecological Model.

## Data Availability

Restrictions apply to the dataset. Data is unavailable due to privacy and ethical restrictions.

## Abbreviations

The following abbreviations are used in this manuscript:

DHET	Department of Higher Education and Training
PA	Physical Activity
NDP	National Development Plan
SDGs	Sustainable Development Goals
SEM	Social Ecological Model
WHO	World Health Organisation
